# Visual Localization across Seasons Using Sequence Matching Based on Multi-Feature Combination †

**DOI:** 10.3390/s17112442

**Published:** 2017-10-25

**Authors:** Yongliang Qiao, Cindy Cappelle, Yassine Ruichek

**Affiliations:** Le2i FRE2005, CNRS, Arts et Métiers, UBFC, Université de technologie de Belfort-Montbéliard, Belfort 90000, France; cindy.cappelle@utbm.fr (C.C.); yassine.ruichek@utbm.fr (Y.R.)

**Keywords:** visual localization, sequence matching, multi-feature combination, place recognition, binary features

## Abstract

Visual localization is widely used in autonomous navigation system and Advanced Driver Assistance Systems (ADAS). However, visual-based localization in seasonal changing situations is one of the most challenging topics in computer vision and the intelligent vehicle community. The difficulty of this task is related to the strong appearance changes that occur in scenes due to weather or season changes. In this paper, a place recognition based visual localization method is proposed, which realizes the localization by identifying previously visited places using the sequence matching method. It operates by matching query image sequences to an image database acquired previously (video acquired during traveling period). In this method, in order to improve matching accuracy, multi-feature is constructed by combining a global GIST descriptor and local binary feature CSLBP (Center-symmetric local binary patterns) to represent image sequence. Then, similarity measurement according to Chi-square distance is used for effective sequences matching. For experimental evaluation, the relationship between image sequence length and sequences matching performance is studied. To show its effectiveness, the proposed method is tested and evaluated in four seasons outdoor environments. The results have shown improved precision–recall performance against the state-of-the-art SeqSLAM algorithm.

## 1. Introduction

In many applications, it is crucial that a robot or vehicle localizes itself within the world especially for autonomous navigation and driving. Since visual sensors are widely used in the robotic community, visual localization based on place recognition plays a key role in visual Simultaneous Localization and Mappings (vSLAM) systems [1,2]. In this context, identifying previously visited locations in a long-term period under environment changes is a big challenge [3,4].

There have been lots of visual localization methods by regarding place recognition as a problem of matching a sensed image against a previously acquired image database in which locations are represented by images. One can cite the FAB-MAP algorithm [5], which employs a Bag-of-Words (BOW) image retrieval technique and a Bayesian frame-work [6] that achieves robust image matching. Another one is SeqSLAM [7], which adopts sequence matching rather than single image matching for place recognition, which achieves significant performance improvements than FAB-MAP. However, in [8], some weaknesses of SeqSLAM were reported, such as the field of view dependence and the complexity of parameter configuration. For these reasons, the community continues searching for new methods that can satisfy the high requirements needed to achieve robust life-long visual localization.

Recently, local binary descriptors that encode patch appearance using compact binary string with low memory requirements are widely used in image description and visual recognition [9,10]. One typical binary descriptor is LBP (Local Binary Pattern) [11], then followed by its new variation–CSLBP (Center-Symmetric Local Binary Patterns) [12]. Their advantages are that they are invariant to monotonic changes in gray-scale and fast to calculate. Nevertheless, as the author [13] noted, local binary descriptors lack spatial and shape information. To overcome this limitation, we propose adding GIST features [14] that focus more on the shape of scene itself and on the relationship between the outlines of the surfaces and their properties. GIST and LBP can be seen as complementary for image representation in the sense that GIST focuses more on global information while local binary descriptors emphasize local texture information.

**Figure 1:**
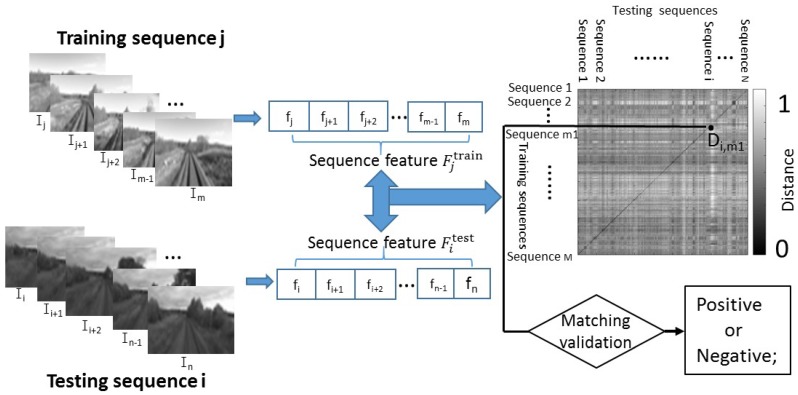
General diagram of visual localization system using sequence matching.

In this paper, the problem of visual localization across seasons is addressed using feature combination (CSLBP and GIST) and sequence matching. Feature combination of CSLBP and GIST is used to make place descriptions more robust, and sequence matching rather than single image matching is used to improve the place recognition ability. Figure 1 illustrates the general diagram of our approach. Based on features extracted from images, sequences are efficiently matched using Chi-square distance and the best candidate matching location is recognized according to coherent sequence matching. Image descriptor used in this paper is a combination of CSLBP and GIST, which should improve image distinguishing ability by capturing local and global image information. The algorithm performance using multi-season videos of 30,000 km long train ride in the northern Norway will be demonstrated. For this, an extensive experimental study is conducted according to sequence matching length and compared the performance of the proposed approach with the state-of-the-art SeqSLAM [7] method.

The paper is organized as follows: Section 2 provides background and related works on visual place recognition. Section 3 details experiment setup: the used dataset and evaluation method. Experiments are presented with results in Section 4. Finally, Section 5 discusses the outcomes of this paper and presents future work.

## 2. Background and Related Works

With cameras widely used in many robot platforms, a large number of vision-based place recognition and localization algorithms have been proposed [15]. Here, we briefly discuss some of the key vision-based localization algorithms proposed over the past decade. FAB-MAP [5] method matches appearance of the current scene image to a reference one by employing BOW (Bag-of-Words) image retrieval system. The SIFT (Scale-Invariant Feature Transform) and SURF (Speeded Up Robust Features) features used in this system allow invariance to scale and rotation, but lead to poor performance across large lighting variations and scene appearance changes.

Another popular method is SeqSLAM [7], which is adopting sequence matching rather than single image matching for place recognition that achieves significant performance improvements than FAB-MAP. The usage of sequences allows higher robustness to lighting or extreme perceptual changes. In SeqSLAM, image similarity is evaluated using the sum of absolute differences between contrast enhanced and low-resolution images without the need of image keypoint extraction. However, in [8], some weaknesses of SeqSLAM were reported, such as the field of view dependence and the complexity of parameters configuration.

In [16], RatSLAM was proposed as a computational model based on competitive attractor networks. This method has been demonstrated in a number of experiments for mapping large environments or over long time periods [17]. Recently, RTAB-MAP (Real-Time Appearance-Based Mapping) [18], based on a long-term memory methodology has been also proposed. In [19], a multi-scale bio-inspired place recognition illustrated how to achieve better place recognition performance by recognizing places at multiple spatial scales.

In recent days, binary descriptors, with their high efficiency (low computational cost) and effectiveness, have been widely used in image description, fast image matching and visual place recognition [20]. [21] proposed a novel approach ABLE (Able for Binary-appearance Loop-closure Evaluation) based on binary codes and disparity information using stereo images. The proposed method applies the LDB (Local Difference Binary) descriptor on a gray-scale image and disparity map, which can decrease the perceptual aliasing influence. Winters et al. [22] utilized an omni-directional camera to create a topological map. The large image set is compressed using PCA (Principal Component Analysis) to form a low-dimensional eigenspace. Then, a robot could determine its global topological position using an appearance based matching method. In [23], the ABLE-M approach proposed resides in the description of sequences of monocular images as binary codes, which are extracted from a global LDB descriptor and efficiently matched using FLANN (Fast Library for Approximate Nearest Neighbors) for fast nearest neighbor search. In [24], the proposed method combines individual strengths of the high-level and mid-level feature layers from a convolutional neural network to partition the search space and recognize places under severe appearance changes.

In addition, some other place recognition methods on mobile platforms are also proposed. In [25], a crowdsourcing-based mobile location recognition system (CrowdLR) was proposed, which leverages a variety of sensors embedded in smartphones to collect richer location fingerprints. The used object-centric fingerprint searching method can achieve higher recognition accuracy. In [26], a method LRACI (Location Recognition algorithm for Automatic Check-In applications) was introduced. Experimental results have shown a location recognition accuracy of about 90%, opening the door to real LRACI employments.

Nowadays, one of the most fashionable and challenging topics in life-long visual topological localization is to recognize previously visited places along different seasons. The method presented in [3] approaches visual topological localization over seasonal conditions by using SURF descriptors [27,28]. Another very recent proposal focused on the prediction of changes on places based on superpixel vocabularies [29], which has been tested using 3000 km dataset across all four seasons.

## 3. Proposed Visual Localization Approach

**Figure 2:**
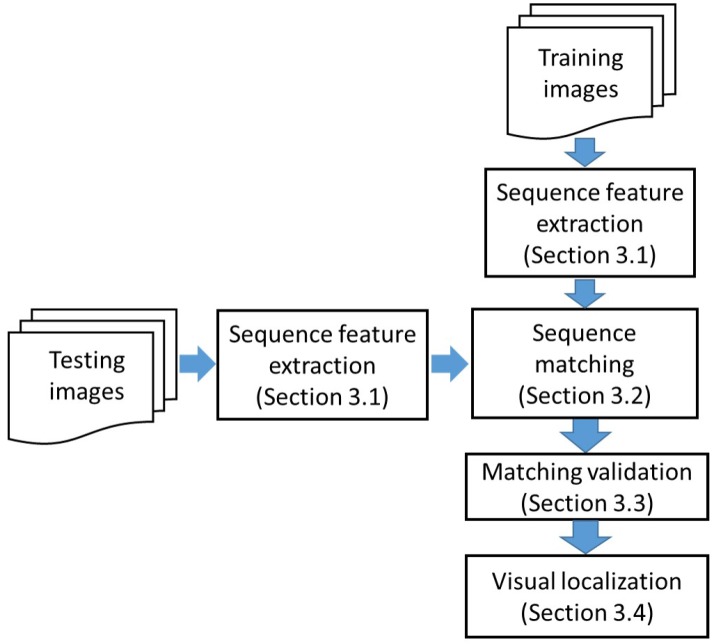
Flow chart of proposed visual localization using sequence matching.

In order to make the place recognition more robust, the proposed method uses CSLBP and GIST features to describe the place. Then, visual localization across seasons is realized based on sequence matching. As illustrated in Figure 1 and Figure 2, there are four main components in our approach: sequence feature extraction, sequence matching, matching validation and the last is visual localization based on the matching result.

To provide detail, a set of GPS tagged training images is firstly acquired. After image preprocessing, CSLBP and GIST features are extracted independently from the images of the training database and then concatenated together to form multi-feature CSLBP++GIST. Then, multi-feature CSLBP++GIST obtained from images of a sequence are concatenated (++) to form the final sequence feature (*F*) representing the sequence (Section 3.1). Here, the sequence consists of consecutive images and each sequence is independent. A current place (represented by a testing sequence) is then recognized through sequence matching step based on Chi-square distance (Section 3.2).

In the step of sequence matching, for each testing sequence, the sequence candidate from the training database that provides the minimum distance can be considered as the most similar one to the testing sequence. In fact, the two best sequence matching candidates are conserved for further verifying the final matching result.

Effectively, the best matching candidate will be validated through a distance ratio SS (Section 3.3), computed from the two minimum scores (the first minimum distance divided by the second minimum distance). If the ratio SS is lower than or equal to a threshold Th, the first best sequence candidate (with the lower distance) is confirmed and regarded as positive matching. Otherwise, it is considered as negative one (in this case, no matching result is conserved). When a sequence candidate is confirmed as positive, the position can be obtained from the GPS information that correspond to the matched training images (Section 3.4).

### 3.1. Sequence Feature Extraction

As mentioned in [30], high resolution images are not needed to perform an effective visual recognition over time. Indeed, high resolution images increase computational cost without bringing significant visual recognition improvement. For image storage and efficient matching, in this work, the original images are down-sampled into 32 × 32 pixels before feature extraction.

Sequence feature extraction consists of four steps: (1) CSLBP feature extraction from single image; (2) GIST feature extraction from single image; (3) then, extracted CSLBP and GIST features are concatenated (++) together to form multi-features; (4) finally, the CSLBP++GIST multi-features obtained from images of a sequence are concatenated (++) to form the final sequence feature (*F*) representing the sequence.

(1) CSLBP feature extraction: As already described, CSLBP is a modified version of LBP. It is closely related to the gradient operator because it compares the gray levels of pairs of pixels in centered symmetric directions instead of comparing the central pixel to its neighbors. In this way, CSLBP features take advantage of the properties of both LBP and gradient based features. For an even number *P* of neighboring pixels distributed on radius *R* (around central pixel ($x_{c}$, $y_{c}$), the CSLBP operator produces $2^{P/2}$ patterns as follows:

(1) $$CSLBP_{P,R}\left(x_{c},y_{c}\right)=\sum_{i=0}^{(P/2)-1}s(|g_{i}-g_{i+(P/2)}\left|\right)2^{i},$$

(2) $$s\left(x\right)=\left\{\begin{array}{cc} 1,\;\; & x>T,\\ 0,\;\; & otherwise, \end{array}\right.$$

where $g_{i}$ and $g_{i+(P/2)}$ correspond to the gray values of center-symmetric pairs of pixels (*P* in total) equally spaced around central pixel ($x_{c}$, $y_{c}$). *T* is used to threshold the gray-level difference so as to increase robustness of CSLBP feature on flat image regions. Since the gray levels are normalized in [0,1], the authors of [12] recommend using a small value for *T*.

For an image of size 32×32, after computation of CSLBP patterns for each image pixel, a histogram is built to represent the image texture:

(3) $$f_{CSLBP}=\sum_{i=1}^{32}\sum_{j=1}^{32}f\left(CSLBP_{P,R}\left(i,j\right),l\right),\;l=0,1,2,3,\cdots ,2^{P/2}-1,$$

(4) $$f\left(x,y\right)=\left\{\begin{array}{cc} 1,\;\; & x=y,\\ 0,\;\; & otherwise. \end{array}\right.$$

Finally, the length of a histogram resulting from the CSLBP feature is $2^{P/2}$. It is obvious that CSLBP produces a shorter feature set than LBP (the feature dimension of LBP is a P∗(P−1)+3).

In this work, eight sampling points and a 3-pixel radius around the center pixel are set. Thus, 16-dimensional CSLBP features are obtained. In terms of the LBP feature (uniform pattern), it is 59 dimensions.

(2) GIST feature extraction: GIST is a global image feature, which characterizes several important statistic information about a scene [31]. A variety of experimental studies have demonstrated that humans perform rapid categorization of a scene by integrating only coarse global information that can be extracted using “ GIST ” [14]. Using the model proposed by Oliva [32], GIST features are computed by convolving an oriented filter with down-sampled images (32 × 32) at several different orientations and scales. The scores for the filter convolution at each orientation and scale are stored in an array and result in a 512-dimensional feature.

(3) Feature combination: After CSLBP feature $f_{LBP}$ and GIST feature $f_{GIST}$ are extracted from an image, respectively, they are combined into a new CSLBP++GIST feature *f*. The combination is simply concatenating (++) the two features:

(5) $$f=f_{CSLBP}++f_{GIST}.$$

Concatenating CSLBP and GIST features can take advantage of local and global image information simultaneously, and thus make the places representing each location more comprehensively.

**Figure 3:**
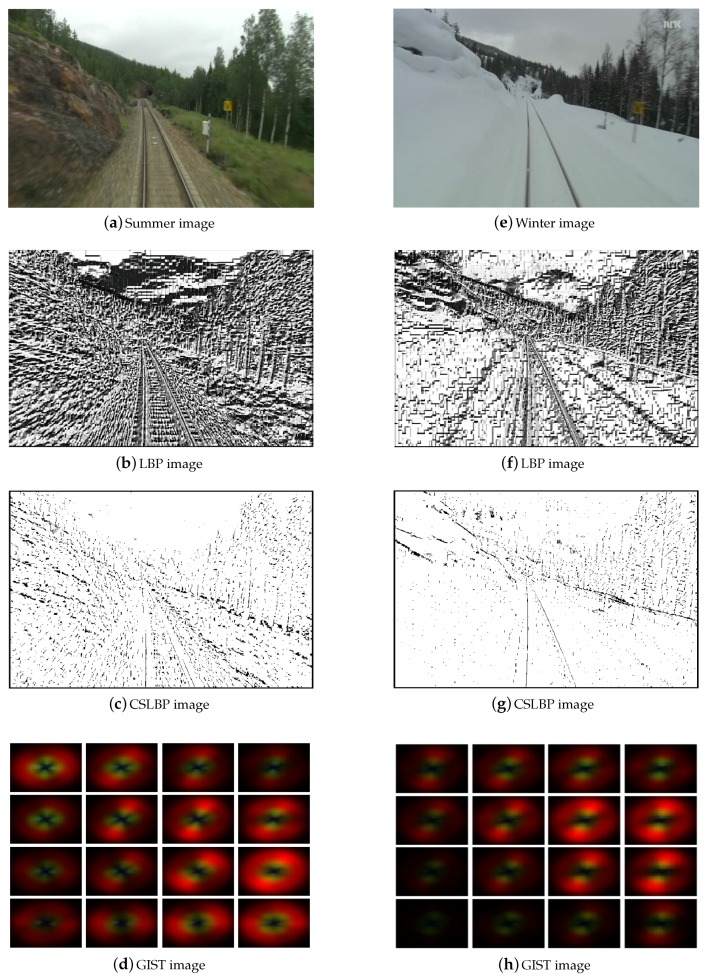
Example of extracted features. The first row shows the original images. The second and third rows show the images of LBP and CSLBP features, respectively. The fourth row gives the images of GIST features.

Figure 3 illustrates an example of extracted features. It can be seen that CSLBP and LBP features pay more attention to the image detail (local information). It also can be found that there are more noises in the LBP image than the CSLBP image. Considering that CSLBP compares the gray levels of pairs of pixels in centered symmetric directions, it can represent the image better than LBP (not sensitive to noise). In terms of GIST features, it shows the global information of the whole scene. Therefore, combination of the local feature—CSLBP—and global feature—GIST—will describe the place better and should improve place recognition performance.

(4) Sequence feature: Finally, the CSLBP++GIST features extracted from each image of a sequence are concatenated (++) to form the final sequence feature (*F*) representing the sequence:

(6) $$F=f_{i}++f_{i+1}++f_{i+2}++\ldots ++f_{m-2}++f_{m-1}++f_{m},$$

where *i*, i+1, ⋯, *m* are indexes of the consecutive images in the sequence, and $L_{length}=m-i+1$ is length of the sequence. The feature dimension of each sequence is 528 × $L_{length}$, and 528 is the sum of 16 (dimensions of CSLBP feature) and 512 (dimensions of GIST feature).

In our work, the original image database is simply divided into sequences with the same length, and each sequence is composed of consecutive images. Since training and testing routes are traveled at a similar speed, the same sequence length is used for the training and testing sequences. Therefore, each sequence can be represented using sequence feature (*F*) with the same dimension.

### 3.2. Image Sequence Matching

To perform sequence matching, similarities between sequence features are evaluated through chi-squared distance. The chi-squared distance is a nonlinear metric that can be calculated easily. Suppose the numbers of training and testing images are $N^{train}$ and $N^{test}$, respectively, and sequence length is $L_{length}$. Therefore, the training sequence number is $M=N^{train}/L_{length}$ and the testing sequence number is $N=N^{test}/L_{length}$.

Given a testing sequence $Q_{i}^{test}$ (composed of the last $L_{length}$ consecutive testing images), it will be compared with each training sequence $Q_{j}^{train}\left(j=1,2,\cdots ,M\right)$ from the training database. Since the sequence lengths are same, the sequence matching can be conveniently conducted using the chi-squared distance.

The similarity value between the two sequences $Q_{i}^{test}$ and $Q_{j}^{train}$ is measured using the chi-squared distance $D_{ij}$, computed as follows:

(7) $$D_{i,j}=\chi^{2}\left(F_{i}^{test},F_{j}^{train}\right)=\sum_{k}\frac{((F_{i}^{test}{)}_{k}-(F_{j}^{train}{)}_{k}{)}^{2}}{|(F_{i}^{test}{)}_{k}+(F_{j}^{train}{)}_{k}|},$$

where $F_{i}^{test}$ is the feature vector of the current testing sequence $Q_{i}^{test}$, and $F_{j}^{train}$ is the feature vector of a training sequence $Q_{j}^{train}$ (from the training dataset). *k* is an index of the components of feature vector: $k=1,2,\cdots ,528\times L_{length}$. Then, all the computed $D_{i,j}$ form a distance matrix *D*.

For sequence matching, the feature vector of the current sequence $Q_{i}^{test}$ is compared with the feature vector of each training sequence $Q_{j}^{train}\left(j=1,2,\cdots ,M\right)$. Based on the distances $D_{i,j}\left(j=1,2,\cdots ,M\right)$, the two best training sequence candidates (which have the first minimum distance and second minimum distance) that match the current testing sequence are conserved.

### 3.3. Matching Validation

In order to reduce false matching cases, the ratio $SS_{i}$ computed from the first minimum distance and second minimum distance is used to validate the matching result (as for single sequence matching):

(8) $$SS_{i}=\frac{D_{i,m1}}{D_{i,m2}},$$

where $D_{i,m1}$ and $D_{i,m2}$ are, respectively, the first and second minimum distances between the feature vector of current testing sequence $Q_{i}^{test}$ and the feature vectors of all the training sequences $Q_{j}^{train}\left(j=1,2,\cdots ,M\right)$ as follows:

(9) $$\left\{\begin{array}{c} D_{i,m1}=\min_{j}\left\{D_{i,j}\right\},\\ D_{i,m2}=\min_{j(j\neq m1)}\left\{D_{i,j}\right\}. \end{array}\right.$$

The values of $SS_{i}$ are between 0 and 1. A threshold is then applied to the score $SS_{i}$ to determine if the sequence pair (i,m1) is matched or not. The matching is considered as positive when the distance ratio $SS_{i}$ is lower than or equal to the threshold Th; otherwise, it is considered negative and the sequence candidate is ignored.

### 3.4. Visual Localization

After one sequence matching candidate is successfully validated, the vehicle can localize itself through the GPS information attached to the matched training sequence. Effectively, since the training images are tagged with GPS or pose information, the vehicle can get its position through the training images that match with the current testing sequence. This is a topological level localization, that is, the system simply identifies the most likely location. Therefore, this is not a very accurate localization because the training and testing trajectories are not exactly same.

### 3.5. Algorithm of Proposed Visual Localization

Algorithms 1 and 2 illustrate the proposed method for sequence matching based visual localization. It includes feature extraction and combination, image sequence matching, matching validation and visual localization steps. Algorithm 1 shows how conduct sequence matching using feature CSLBP++GIST, while Algorithm 2 gives the matching validation method and final visual localization results.
**Algorithm 1** Sequence matching using feature CSLBP++GIST.**Inputs:**  ${\left\{I_{j}^{train}\right\}}_{j=1}^{N^{train}}$ {training images}; ${\left\{I_{i}^{test}\right\}}_{i=1}^{N^{test}}$ {testing images };  $N^{train},N^{test}$ {training and testing images numbers}; $L_{length}$ {Sequence length};**Outputs:**  D{distance matrix};**Algorithm:**  /∗ TRAINING PHASE ∗/  **for**
*j*← 1 to $N^{train}$/$L_{length}$
**do**  $F_{j}^{train}$←$f_{CSLBP}++f_{GIST};$//Feature of training sequence.  **end for**  /∗ TESTING PHASE ∗/  /∗ Sequence matching based on feature sequences; Section 3.2 ∗/  **for**
*i*← 1 to $N^{test}/L_{length}$
**do**  $F_{i}^{test}$←$f_{CSLBP}++f_{GIST};$//Feature of testing sequence.  **for**
*j*← 1 to $N^{train}/L_{length}$
**do**   $D_{i,j}\leftarrow \sum_{k}\frac{{\left({\left(F_{i}^{test}\right)}_{k}-{\left(F_{j}^{train}\right)}_{k}\right)}^{2}}{|{\left(F_{i}^{test}\right)}_{k}+{\left(F_{j}^{train}\right)}_{k}|}$; //*k* is the index of the components of feature vector (See Equation (7)).  **end for**  **end for**
**Algorithm 2** Sequence matching validation and visual localization.**Inputs:**  *i* { Index of testing sequences}; *j* {Index of training sequences };  DD {Distance matrix between the testing sequences and training sequences};**Outputs:**   SS{distance ratio}; Vehicle position**Algorithm:**  **for** Each testing sequence *i* ← 1 to $N^{test}/L_{length}$
**do**  $SS_{i}=\frac{\min_{j}\left\{D_{i,j}\right\}}{\min_{j(j\neq m1)}\left\{D_{i,j}\right\}}$; m1 is the index of the first minimum distance.  if $SS_{i}<=Th$   Matching validation is positive;   Vehicle position ← the matched training image position  if $SS_{i}>Th$   Matching validation is negative;   Vehicle position ← NaN (no position result)  **end for**

## 4. Experimental Setup

### 4.1. Dataset and Ground-Truth

**Figure 4:**
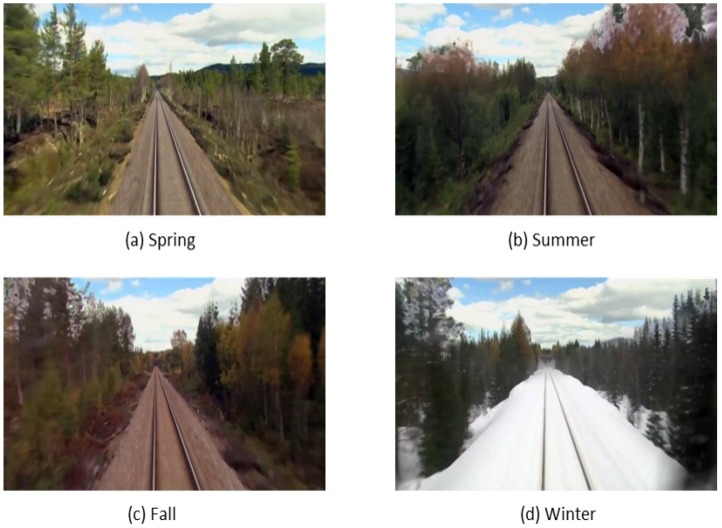
Typical four season images representing the same scene in spring, summer, fall and winter. It can be seen that huge differences appear in the images with seasons changing.

The dataset used in our work is an open dataset called Nordland (https://nrkbeta.no/2013/01/15/nordlandsbanen-minute-by-minute-season-by-season/). It is composed of footage videos of a 728 km long train ride between two cities in north Norway [8]. The complete 10 h journey has been recorded in four seasons. Thus, the dataset can be considered as a single 728 km long loop that is traversed four times. As illustrated in Figure 4, there is an immense variation in the appearance of the landscape, ranging from green vegetation in spring and summer to colored foliage in autumn and complete snow-cover in winter over fresh.

In addition to the seasonal changes, different local weather conditions like sunshine, overcast skies, rain and snowfall are experienced on the long trip. Most of the journey leads through natural scenery, but the train also passes through urban areas along the way and occasionally stops at train stations or signals. The original videos have been recorded at 25 fps with a resolution of 1920 × 1080 using a SonyXDcam with a Canon image stabilizing lens. GPS readings were recorded in conjunction with the video at 1 Hz. The full-HD recordings [33,34] have been time-synchronized such that the position of the train in an arbitrary frame from one video corresponds to the same frame in any of the other three videos. This was achieved by using the recorded GPS positions through interpolation of the GPS measurements to 25 Hz to match the video frame rate.

For the experiments described in the following, image frames are extracted from the original videos at 1 fps, and there are then 35,768 image frames for each season. Each image is then down-sampled to 32 × 32 pixels and converted into gray-level images.

### 4.2. Evaluation Method

Precision-recall characteristics are widely used to evaluate the effectiveness of image retrieval [9]. Therefore, our evaluation methodology is based on precision–recall curves. These curves are determined by varying the threshold Th between 0 and 1, applied to the ratio SS and calculating precision and recall. Precision relates to the number of correct matches to the number of false matches, whereas recall relates to the number of correct matches to the number of missed matches. Positives are considered when the ratio is lower than or equal to the threshold Th. Here, 100 threshold values are considered to obtain well-defined precision–recall curves.

In this experiment, the training image number is equal to the testing image number, and each testing image has a ground truth matching. Therefore, there are only true positives (correct results among successfully validated image matching candidates) and false positives (incorrect results among successfully validated image matching candidates). The sums of the true positives and false positives are the total retrieved image numbers.

## 5. Experiments and Results

### 5.1. Feature Combination Analysis

In a first set of experiments, we evaluate how well feature combinations perform for place recognition and also compare the results with those obtained by the state-of-the-art SeqSLAM method. For the evaluation of SeqSLAM, the source code provided by OpenSeqSLAM [8] is used. For the SeqSLAM parameters setting, the image size is 32 × 32, $V_{min}=0.9$, $V_{max}=1.1$, $V_{step}=0.02$, and the other parameters use the default as reported in [8].

**Figure 5:**
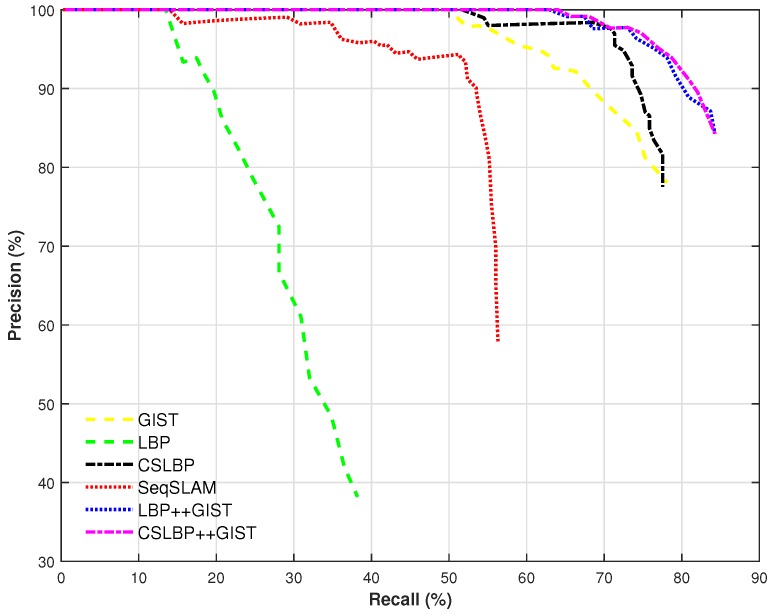
Performance of the proposed method according to different used features and in comparison with the SeqSLAM method (summer vs. winter, sequence length is 200).

The experiments were conducted using the videos presenting an extreme situation in terms of appearance changes (spring vs. winter). The length of each sequence is 200 images. As shown in Figure 5, the proposed method using CSLBP performs relatively well at a high precision level, while GIST outperforms the SeqSLAM method. In general, CSLBP and GIST features perform similar when they are used independently. However, when using the multi-feature (CSLBP++GIST), the retrieval ability is increased significantly. The reason is that CSLBP++GIST takes advantage of local and global information can distinguish the similar images more accurately.

It can be seen that our method with CSLBP++GIST can reach around 65% of recall at 100% precision, which outperforms LBP++GIST a little and the SeqSLAM method significantly. Among these different features, the performance of LBP feature is the worst while the performance of CSLBP++GIST is the best.

### 5.2. Sequence Length Selection

**Figure 6:**
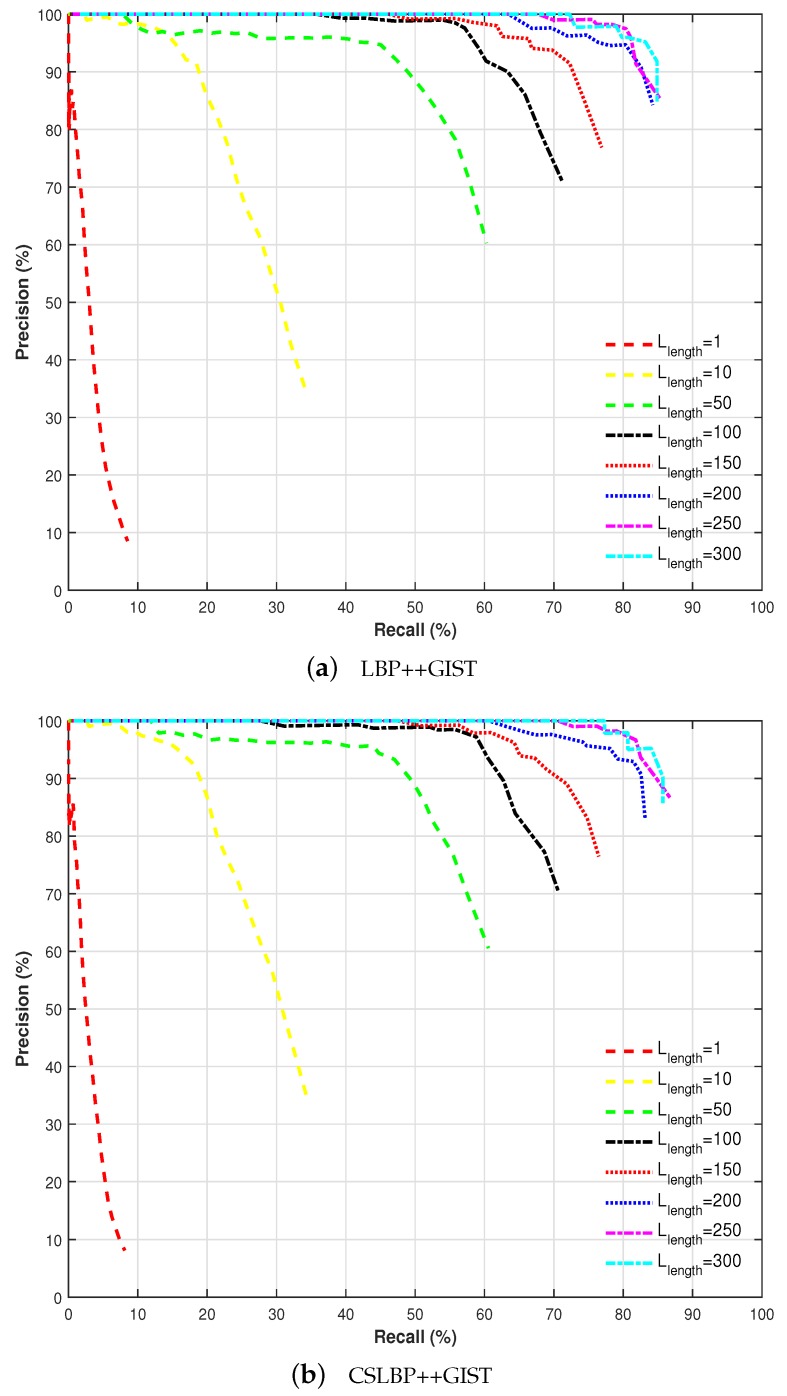
Performance comparison of our proposed method with different feature combination, according to image sequence length $L_{length}$ (spring vs. winter).

Traditionally, visual localization has been performed by considering places represented by single images. Recently, several approaches such as SeqSLAM have proved that recognizing places through sequences of images is more robust and effective [7]. In this work, we also follow the idea of using sequences of images instead of single image for identifying places. This approach allows for achieving better results for visual localization in different seasons, as it can be seen in Figure 6.

Figure 6 shows the performance achieved by varying sequence length from 1 to 300 frames for two different feature combinations: LBP++GIST and CSLBP++GIST. Significant performance improvement is achieved by increasing the sequence length up to 200 frames, after which the improvement became modest.

According to the precision–recall curves demonstrated in Figure 6, the influence of sequence length ($L_{length}$) is decisive for improving the performance of visual localization in different seasons. Moreover, there is a limit near a length of 200 frames, from which the results are not greatly enhanced. For this reason, sequence length $L_{length}$ is set to 200 frames in the rest of the experiments.

### 5.3. Visual Localization under Different Season Couples

After feature performance evaluation and sequence length selection, visual localization using sequence matching based on multi-feature combination (CSLBP++GIST) was compared under different season couples.

**Figure 7:**
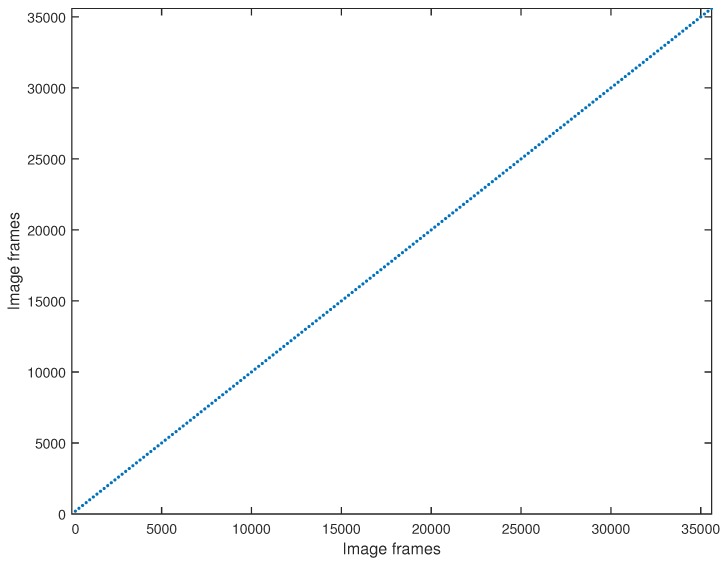
Ground truth. Since the frame from one season corresponds to the same frame in any of the other three seasons, the ground truth is diagonal.

Figure 7 illustrates the ground truth of image matching (for every possible couple of seasons). It should be noted that the position of the train in an arbitrary frame in one season corresponds to the same frame in any of the other three seasons thanks to time-synchronization.

**Figure 8:**
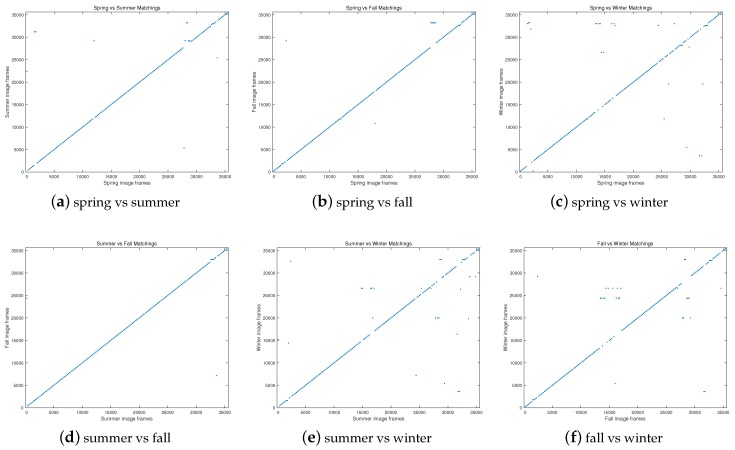
Matching results under different season couples at 100% recall situation. The expected results (true matching) are along the diagonal.

The matching results under different season couples are depicted in Figure 8. As our objective is to correctly identify the place as much as possible (along the diagonal), it can be seen that the result of “summer vs. fall” is the best among the others. It can be also noticed that when the winter sequence is evaluated (Figure 8c,e,f), the number of unrecognized places increase, which is because the snow in winter leads to featureless scenes.

**Figure 9:**
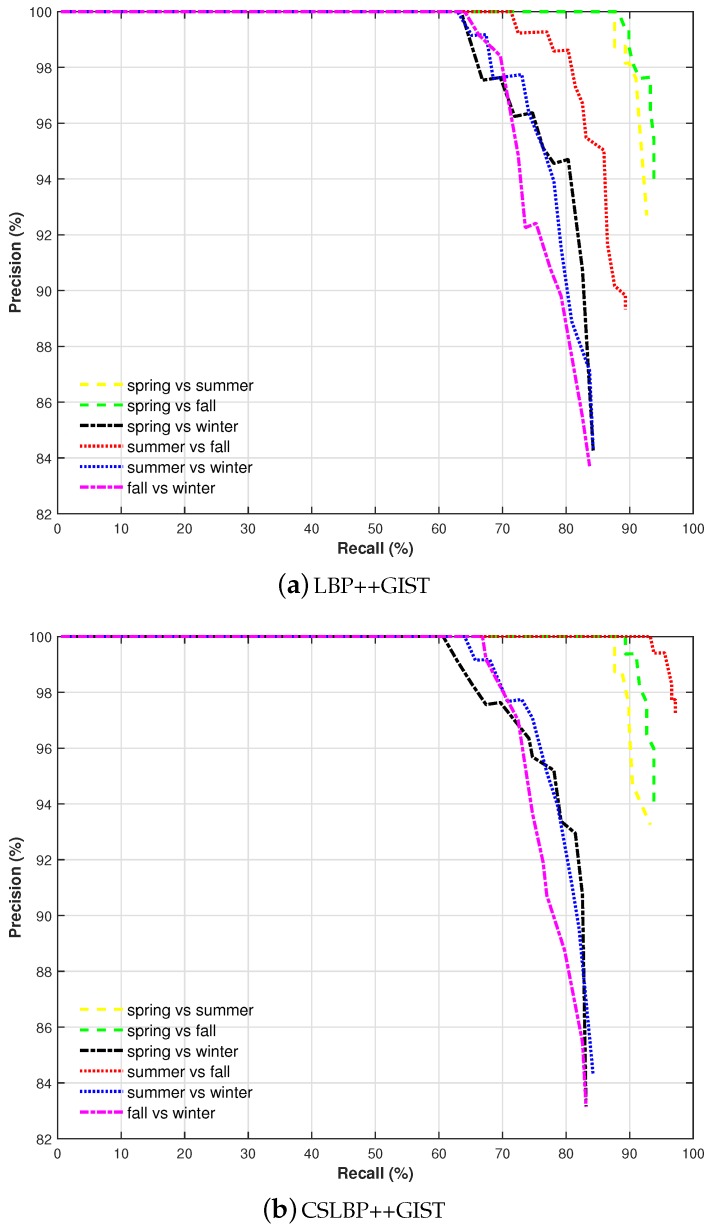
Precision–recall curves comparing the performance of different feature combination under different season couples.

Figure 9 shows precision–recall curves of matching results under different season couples. It can be easily found that visual recognition performance of our method is better under (spring vs. summer), (spring vs. fall) and (summer vs. fall), where we can reach above 85% of recall at 100% precision level. It can be seen also that the proposed multi-feature combination method can achieve recall rate above 60% at 100% precision under all of the season couples. The overall performance of CSLBP++GIST is better than that of LBP++GIST. As expected, when winter sequence is evaluated, the effectiveness of the proposed method decreases due to the extreme changes that this season causes in place appearance because of environmental conditions such as presence of snow, illumination and vegetation changes, etc.

**Figure 10:**
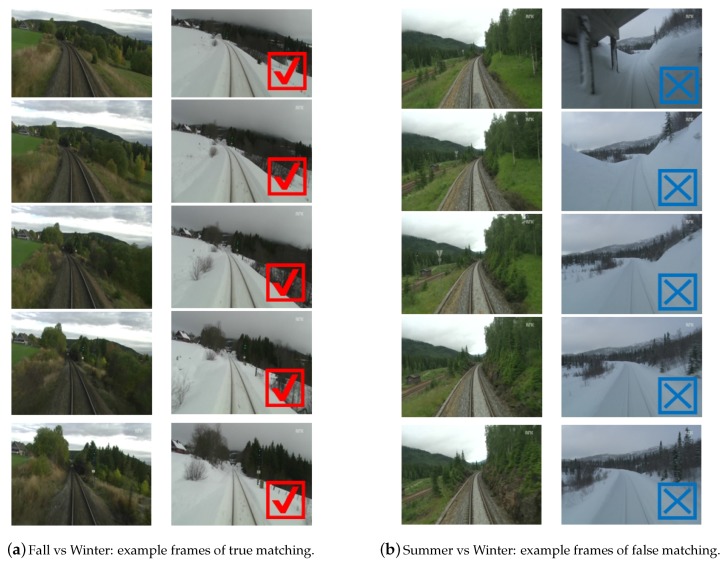
Corresponding frames from sequence matching in two cases (“fall vs. winter” and “summer vs. winter”). In each sub-figure, the left column shows image frames queried from one season traversal, and the right column shows the image frames recalled by our approach.

Figure 10 shows two examples (true and false) of frame matches using the proposed method. True matching frames from “fall vs. winter” can be achieved (seen in Figure 10a), despite appearance variations (many vegetations in fall while snow covering the ground in winter). Through false matching shown in the Figure 10b, it can be seen that the image frames from winter season are almost featureless due to heavy snow cover. Therefore, few false matching cases appeared in this extreme weather situation. According to the experiments’ results, on most occasions, place recognition using the multi-feature attained good matching performance.

**Figure 11:**
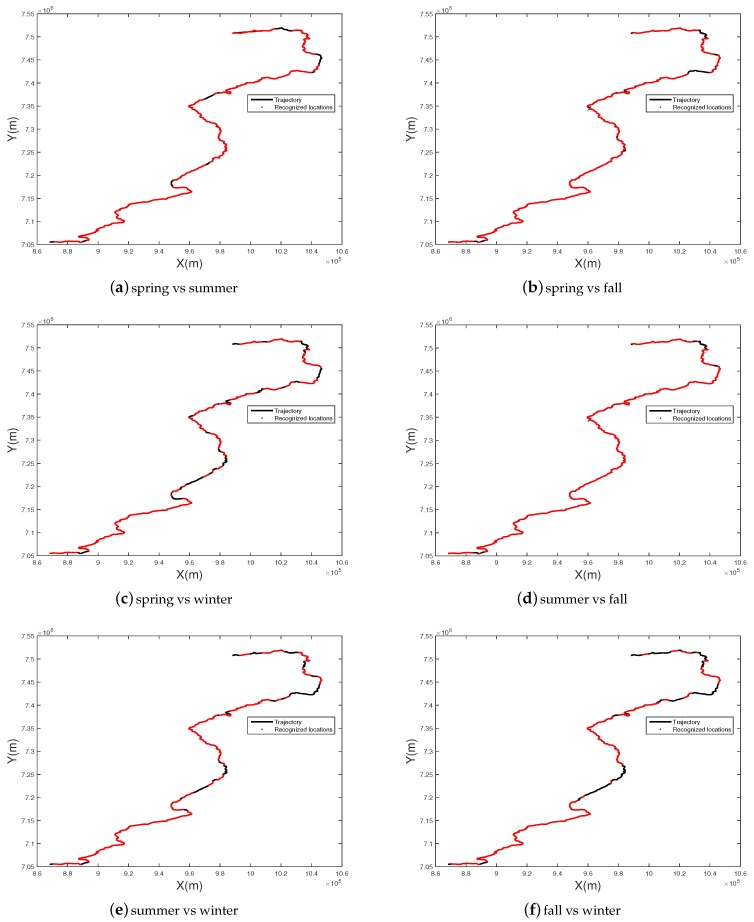
Visual localization results under different season couples at 100% precision level. Successful matched images that come from the same location (on the basis of appearance alone) are marked with red points.

Figure 11 shows visual localization results of different season couples at 100% precision level. It can be seen that most places can be successfully localized, and at least 60% of the places (red points) can be localized in the worse matching case (spring vs. winter).

For the visual localization based on place recognition, we are primarily interested in the recognition rate high precision level. The recall scores for high selected precision values of SeqSLAM and the proposed approach are given in Table 1. As illustrated before, the SeqSLAM parameters setting are as follows: $V_{min}=0.9$ , $V_{max}=1.1$, $V_{step}=0.02$, and the other parameters use the default as reported in [8]. According to Table 1, for most cases, the proposed place recognition based visual localization algorithm achieves the better recall rate. Moreover, in “spring vs. summer” and “spring vs. fall” situations, the recall rates of the proposed approach are higher than 87% for all the high precision values recorded in Table 1.

For both SeqSLAM method and the proposed approach, the recall rate increases when precision is decreasing. The recall rate of the two methods increases drastically at 90% precision. In addition to that, the recall rate of SeqSLAM method is lower than the recall rate of the proposed method, and the worst for all the high precision values. This is probably due to the fact that the SeqSLAM method has a certain dependence on the field of view and the image size, as demonstrated in [8]. The experiment was conducted on an Intel Core i7, 2.40 GHz laptop. Average processing times for each image description are presented in Table 2. The proposed CSLBP++GIST feature for image description is faster than the others. This is because the feature dimension of CSLBP++GIST is lower than that of LBP++GIST, which makes the image description and computation more efficient. In addition, the computational time of sequence matching using the combined feature (CSLBP++GIST) is illustrated in Table 3. The computational time increases when the size of the training database is large. Since the CSLBP++GIST feature dimension is lower than that of LBP++GIST, the processing time of CSLBP++GIST based matching is shorter. Compared with SeqSLAM, the proposed method using CSLBP++GIST feature is faster.

## 6. Conclusions

In this paper, a feature combination based sequence matching method is proposed to perform robust localization even under substantial seasonal changes. After feature extraction, chi-square distance is used to measure similarity between a testing sequence and the training sequences of a training database. A distance ratio is then calculated before applying a thresholding procedure to validate the good matching candidates.

Thanks to precision–recall based evaluation, experimental results showed that the proposed sequence matching method is more robust and effective for long-term visual localization in challenging environments. The proposed method takes advantage of local and global image information, which can reduce the aliasing problem. Sequence length analysis demonstrated that sequences as long as 200 frames could provide viable recognition results. Shorter sequences cannot achieve acceptable results, while longer ones cannot bring significant improvement. Compared to the state-of-the-art SeqSLAM method, the proposed approach provides better recognition performances. In addition, according to the localization results, at least 60% of the places can be localized using the appearance through the proposed method.

However, using feature combination increases feature vector dimension and thus increases time computation. To overcome this limitation, we envision dealing with dimension reduction using space projection techniques or searching methods like local sensitive hashing. Another drawback is that, in the experiments performed, testing and training sequence lengths the same as that of twice the driving speeds of trains are very close. In the future, more flexible sequence length selection and matching strategy should be considered.

## Figures and Tables

**Table 1:** Recall scores at selected high precision levels (100%, 99%, 90%).

Different Season Couples	Method	100% Precision (%)	99% Precision (%)	90% Precision (%)
Spring vs. Summer	SeqSLAM	20.45	27.73	66.11
	LBP++GIST	87.64	87.64	92.70
	CSLBP++GIST	**87.64**	87.64	93.26
Spring vs. Fall	SeqSLAM	15.41	27.45	63.87
	LBP++GIST	88.20	89.89	93.82
	CSLBP++GIST	**89.33**	91.01	93.28
Spring vs. Winter	SeqSLAM	14.29	17.37	62.18
	LBP++GIST	**63.48**	66.58	82.58
	CSLBP++GIST	60.67	62.92	82.58
Summer vs. Fall	SeqSLAM	9.80	23.81	65.27
	LBP++GIST	71.35	76.97	87.64
	CSLBP++GIST	**93.26**	95.51	97.19
Summer vs. Winter	SeqSLAM	14.01	27.45	53.50
	LBP++GIST	62.92	67.42	79.21
	CSLBP++GIST	**64.04**	67.98	80.90
Fall vs. Winter	SeqSLAM	2.24	2.35	44.82
	LBP++GIST	64.40	66.29	77.53
	CSLBP++GIST	**66.85**	67.42	76.97

**Table 2:** Comparison of average describing times for each image (/s).

Methods	SeqSLAM	LBP++GIST	CSLBP++GIST
Time (/s)	0.1327	0.1476	0.1226

**Table 3:** Comparison of average processing times for image matching (/s).

No. Images in Database	SeqSLAM	LBP++GIST	CSLBP++GIST
200	3.5335	3.1280	2.9463
2000	54.6982	20.4664	18.3309
20,000	87.6982	33.4664	37.3309

## Abbreviations

The following abbreviations are used in this manuscript:

FAB-MAP	Fast Appearance Based Mapping
SeqSLAM	Sequence Simultaneous Localisation and Mapping
GIST	The “GIST” is an abstract representation of the scene
CSLBP	Center-Symmetric Local Binary Patterns
SIFT	Scale-Invariant Feature Transform
SURF	Speeded Up Robust Features
